# A Case of Encephaloid Tumor

**Published:** 1848-08

**Authors:** George L. Upshur

**Affiliations:** Norfolk Virginia


### MEDICAL EXAMINER,

AND

### RECORD OF MEDICAL SCIENCE.

NEW SERIES.—NO. XLIV.—AUGUST, 1848.

---

### ORIGINAL COMMUNICATIONS.

---

*A Case of Encephaloid Tumour.* By George L. Upshur, M. D., of Norfolk, Virginia.

Mrs. C——, the subject of this notice, was 73 years old on the 28th of February, 1848; of a sanguineous, lymphatic temperament, and originally robust constitution. She had not a gray hair in her head, and could see to do the finest needle-work without spectacles, having never been *near sighted.* For more than twelve years before her death, she was subject to a discharge from the vagina of a sanious fluid, unaccompanied by pain, or other unpleasent sensation, and apparently without detriment to her general health, which, until April, 1847, had been uninterruptedly good.

About the beginning of April, she felt upon the scalp, just over the left parietal protuberance, an intensely burning, itching sensation over a surface about as large as a shilling. The part soon became red and tumid, and the inflammation extended rapidly over the whole scalp and upper part of the face. Her medical attendant pronounced it a case of erysipelas, and so treated it. In a few days the inflammation subsided entirely, when there was observed upon the spot where the erysipelas first appeared, a small, well defined tumour, about the size of a pea, apparently just under the skin, and very movable. Four weeks from this time—about the middle of May, 1847—the tumor was as large as a marble, and the physician in attendance, taking it for a simple furuncle, made a free incision into it, from which there flowed out only a little blood. The tumour now commenced to grow rapidly, and, by the end of July, was as large as a walnut, and hard like a wen. About this time, she commenced to feel in the part some pain of a lancinating, gnawing character, for the relief of which she was advised to use emollient poultices, which she did, but without benefit.

On the 23d of November, I saw the tumour for the first time, and examined it carefully. It had then attained a very considerable size, being cylindroid in shape, and measuring eleven inches in circumference, and seven inches from side to side across the summit. It was soft at the apex, and elastic to the touch throughout, slightly movable apparently beneath the skin, and covered with hair as the other parts of the head. The patient had lost flesh and strength within the past four months, and was frequently kept awake by pain in the tumour, which extended down the left side of the neck and arm. Her appetite, however, remained as good as usual, and all the functions both of mind and body were well performed.

Dr. Silvester saw the case with me about the first of December, and made very firm pressure upon the tumour without producing any effect upon the brain. He looked upon it as a melicerous lypoma, and recommended its extirpation. On the 9th of December, Drs. Moore, Tatem and Wright saw the case, and examined it carefully. Dr. Wright did not give an opinion. Dr. Tatem thought that it was a non-malignant tumour, encysted, and filled with a semi-fluid matter, that might readily be let out by a puncture. Dr. Moore, who made a more minute examination into the general health of the patient before and after the appearance of the disease, was of the opinion that it was malignant in its character—probably the *soft cancer* of some authors. We all agreed that extirpation offered the only hope of curing the disease.

Dr. Andrews saw the patient on the 11th of December. He agreed with Drs. Silvester and Tatem as regarded the nature of the tumour, and attributed the pain which she suffered to distension of the integuments of the head and pressure upon the adjacent parts;* and advised, that a puncture should be made into the tumour, the contained fluid let out, and pressure exercised upon the part. Should it re-appear it was again to be punctured, and the cyst injected with some stimulating fluid, as in the radical cure of hydrocele.

Accordingly, on the afternoon of the same day, in the presence of Dr. Moore, I made an incision, with a straight bistoury, about half an inch long, on the left side of the tumour, a little below the summit. There appeared a little blood from the cutaneous vessels, and nothing else. Dr. M. then introduced the canula of a small trochar about three inches into the tumour, and moved it about in every direction. Slight pressure upon the sides ejected, with considerable force, several lobules of semi-soft matter, taken at the time for fat, and a considerable amount of blood. The wound was now closed without further exploration, the patient having lost about four ounces of blood.

Upon washing the pieces discharged, we found that they were not fatty, but fibrous in some points, and resembled cerebral matter in others.

The tumour being now ascertained positively to be malignant, I determined to state fairly the advantages and dangers of an operation for its removal, to the patient, and to give her the election. Having told her, that the tumour, if permitted to remain, would certainly destroy life in a short time, and that the remnant of her days would be passed in great pain—while on the other hand, she might perish under the operation, or from the effects of it; and after all, if extirpated, the tumour might return either in the same place or elsewhere;—she replied, "I would prefer to die under the knife rather than to suffer what I have suffered for the past ten days—I want it cut out."

I now determined, in order to ward off as far as possible the shock of the operation, to administer the vapor of sulphuric ether, it having been ascertained from several previous trials that the patient could be easily brought under its influence, and could be kept so for at least one hour without unpleasant effects.

* I am thus minute in giving the opinions of the medical gentlemen who saw the case, in order to show how much difficulty was attendant upon making a correct diagnosis. Upon this fact I base in part the propriety of the subsequent operation.

On the 18th of December, the patient was placed in a recumbent position, and the ether administered by Mr. T. G. Clayton, dentist. For the first five minutes it had an excitant effect, quickening the pulse, and causing the patient to laugh immoderately. In the course of five minutes more, the pulse became slower, without loss of volume, and the patient was fully under the influence of the agent.

In the presence of Drs. Andrews, Moore and Tatem, (being assisted by the two latter) and Mr. T. W. Upshur, medical student, I commenced the operation by making two elliptical incisions across the tumour antero-posteriorly. Having dissected off the scalp as far as the base of the tumour on either side, an effort was made to tear it up from the base with the handle of the scalpel, but it adhered to the pericranium so closely that this membrane seemed to form the base of the cyst. It was dissected up from the skull, the bone beneath, over the entire surface upon which the tumour rested, being as rough as if it had been rasped. Just in the centre of the base, for an inch and a half in diameter, both tables of the skull were gone, and the cyst had passed through the opening and adhered to the dura mater. The brain could be plainly seen pulsating through the hole, the edges of which were very rough.

About eight ounces of blood were lost during the operation, chiefly from branches of the temporal and occipital arteries. There was so much oozing of blood from the opening in the cranium that it was impossible to distinguish the portion of the cyst which rested upon the dura mater from that membrane itself, and therefore no effort was made to detach it. The wound was dressed by filling it with charpie, applying a compress over it, and covering the whole with a spica bandage, drawn sufficiently tight to arrest the hemorrhage.

As the last turns of the roller were applied, the sponge was taken from the patient's nose. In a few moments, she came to herself, and said to me, "Do not put my cap on until you have taken the tumour off, for I have determined to have the operation performed to-day, and I want it done." She was surprised when told that the tumour was off, and would not believe it until assured of the fact by her grandson, who was present. She was under the influence of the ether one hour and a quarter. Three hours after the operation, the pulse was perefctly natural, and the patient rested better the following night than she had done for six months.

On the 24th of December the dressings were first removed. The wound exhaled a very fetid odour, but exhibited several healthy granulations at its edges. The perforation was completely filled up with encephaloid matter and coagula, beneath which the pulsation of the brain, synchronous with the pulse, was very perceptible. A portion of this matter was torn off, when hemorrhage ensued, and it was deemed prudent to re-apply the dressings. They were lighter than in the first instance.

On the 26th, again dressed the wound. It was still very fetid, and discharged a sanious pus. Applied to it a solution of creasote. 28th.—Perforation filled with a fungous growth, lobulated, rising above the surface of the skin and bleeding freely upon being punctured or roughly handled. The edge of the skin next to the sagittal suture was partially everted and unhealthy in its appearance, and there was a portion of cancerous matter at the anterior part of the wound, running gradually into the healthy granulations on one side, and somewhat overlapped by the skin on the other. The edge of the wound next to the temple was very healthy. Applied sulphate of copper to every part of the diseased mass.

On the 29th I removed a portion of the fungus as large in extent as a shilling, and applied powdered sulphate of copper to every part where there were remains of the cyst. The patient's appetite, strength and spirits remained good.

On the 5th of January the fungus had attained the size of the bottom of a tumbler, and being extremely vascular, an effort was made to coagulate the blood in the nourishing vessels by plunging heated needles in every direction through the base of the fungus. This seemed, however, to exercise but little influence over its growth.

On the 13th I passed a common thread ligature around the base of the fungus, and twisted it off without any difficulty. A surface was thus exposed as large almost as the original wound, which bled very freely. The hemorrhage was arrested promptly by the sulphate of copper in powder. The patient suffered but little pain in the wound, and had not lost strength or flesh since the operation.

From this time until the 9th of March, the wound was dressed every second day, and at each dressing I removed with the scalpel a portion of the fungus as large as a dollar, and applied to the cut surface the actual cautery, and over the whole mass, concentrated nitric acid. I was thus enabled at every dressing to remove with the forceps a considerable slough; so that the amount removed, by scalpel and forceps, was more than equal to the increase. On the 9th of March the appearance of the wound was cupped, the bottom of the depression being one inch and a quarter below the healthy surface of the head, and surrounded by a margin of healthy granulations mingled with small fungi of encephaloid matter. There was very little vascularity in any portion of the wound, and the patient was stronger and in better spirits than usual. I filled up the depression with melted wax, so as to permit the acid to remain upon the margins; and left the patient with a slight hope that at length the morbid growth was somewhat under my control. Up to this time, absorption of the bone had been going on steadily, so that the diameter of the perforation was at least two inches.

Early on the morning of the 10th of March, I received a message that the patient was dying. Found her senseless, with a labored respiration, slow, feeble pulse, and partial paralysis of the right side. These symptoms came on without premonition. Thinking that probably the bandage was so tight as to produce pressure upon the brain, I took off the dressings, but with no amelioration in the symptoms. She remained in this condition twenty-four hours, when there were signs of returning consciousness. Gradually she regained the use of the right side, so that on the 15th she was entirely rational, and in as good condition, to all appearances, as before the advent of these anomalous symptoms—with the exception of a partial loss of control over the tongue, which at times prevented her from making herself understood, and which continued until the 1st of April. During the interval, from the 10th to the 15th of March, the condition of the patient was such, that no attempt was made to remove portions of the fungus. The dressings were changed simply from motives of cleanliness.

From this time, the fungus seemed to be endowed with new life, and its growth was rapid beyond conception. During the five days before mentioned, the depression was completely filled up, and the whole diseased mass was more vascular than I had ever seen it. About the middle of April I excised a piece as large as a shilling, and there was a jet of blood from an artery the "size of a crow quill," besides a rapid effusion of blood from several smaller vessels, to arrest which, the actual cautery, sulphate of copper, nitric acid, and compression were successively resorted to. This condition of the fungus, coupled with the fast failing strength of the patient, deterred me from a further resort to the knife, and I was forced to content myself with passing ligatures around different portions of the mass, and gradually tightening them at each dressing, so as to produce in the end sufficient strangulation to make them slough.

May 1st.—Up to this time the patient had taken various tonic and alterative medicines, among which were the chalybeates and sarsaparilla, but without perceptible benefit. The lemon colour of the skin, so characteristic of cancerous disease, was well marked, and she commenced to suffer considerable pain of a lancinating kind through the temples and around the tumour. The lymphatic glands on the left side of the neck also became enlarged and painful. Her appetite still remained good, but her spirits were flagging, and she had lost flesh and strength since the first of February. The fungus was circular, and extended from the crown to the upper part of the ear anteriorly, and to the root of the neck posteriorly. It was lobulated about two inches above the healthy surface, and discharged a large amount of muddy serum mixed with blood,—so as to saturate several layers of thick cotton cloth in twenty-four hours.

Throughout the entire course of the disease, there was exhaled from the fungus a most intolerable odour. I do not know how I can better describe it than to say, in the quaint language of a professional friend, who saw the case frequently, that "it was a *violent* stink." To control this, creasote, pyroligneous acid, and chloride of soda were freely used, but with little or no effect. The creasote was most effectual.

On the 15th of May I discovered, for the first time, maggots in the tumour, upon which, nitric acid, and a solution of sulphate of copper, and pyroligneous acid had little or no effect. At the next dressing, I applied powdered camphor to the part, after which I saw no more of the worms, except now and then two or three in places where it was difficult to apply the camphor. The camphor also seemed to lesson the fetor to a considerable degree.

About this time partial hemiplegia of the right side supervened, the mind being perfectly clear. Diarrhœa came on about the 1st of June; the appetite failed suddenly, and the patient died on the 10th of the month, having retained her consciousness up to the last moment.

*Autopsy fourteen hours after Death.*

*General Appearance.*—Countenance tranquil; great emaciation; body would not weigh more than 100 pounds. The straw colour of the skin well marked.

The bandages being removed from the head, there appeared a soft, lobulated vascular mass, interspersed with fibrous septa, and weighing about two pounds. It was circular, about eight inches in diameter, and, from its own weight and decomposition of its fluids, was readily separated with a little dissection.

Beneath this fungus and forming its base, was seen a mass of cerebriform, fatty looking matter, which occupied the perforation in the bone and extended beyond its margin, thus shutting out from view the dura mater and brain.

Having made a lateral incision through the scalp from ear to ear, and turned down the flaps behind and before, a view of the calvarium was obtained, which brought out in higher relief the cerebriform mass alluded to above. Around the perforation was a circular sulcus or depression produced by absorption of the bone, the result of pressure of the tumour. This depression was deepest on the posterior superior margin, just where the largest portion of the tumour rested.

Upon removing the calvarium, a full view of the opening in the parietal bone was obtained; this opening was elliptical in shape, 3 by 2½ inches in extent, with margin serrated and thinned by absorption. Just beneath, and contiguous to the perforation, the dura mater was thickened, diaphanous, slightly injected; and the great middle arteries were deeply imbedded in the parietal bone, and were much larger than natural. They, no doubt, contributed considerably towards the nourishment of the tumour. The diploë, as seen upon the edges of the opening, was completely saturated with true encephaloid matter.

The dura mater was now lifted off, and on its inferior side, immediately in the axis of the original tumour, a perforation was found, measuring in extent one inch by one inch and a half. Just beneath the dura mater, and on each side of the perforation, there was a nascent, lardaceous cerebriform tumour, about the size of a walnut, surrounded by tumours of the same kind, five or six in number, and varying in size from a small pea to a filbert. These masses were half imbedded in the posterior lobe of the left hemisphere, the convolutions of the brain overlapping the two large ones on either side a good deal like the anterior portion of the left lung overlaps the heart. They were attached to the brain by very fine, delicate, cellular tissue, and being lifted from their beds, adhered loosely to the under side of the dura mater.

The pia mater and arachnoid, contiguous to this portion of the brain, were slightly injected, but there was neither rupture nor erosion of them, nor was there the slightest alteration of the cerebral substance beneath the tumours. The convolutions at the bottom of the depressions were as distinct as in any other portion of the brain. The cause of the hemiplegia was completely demonstrated by the partial invagination of these masses, and the consequent displacement of the cerebral lobe.

The brain generally was in a healthy condition, except a more than ordinary fulness of the venous system, and a deposition of minute white points along the longitudinal sinus supposed to be concretions of lymph, or encephaloid matter.

It is to be regretted that want of time prevented us from examining the thorax and abdomen. I doubt not that cancerous matter would have been found in the viscera of both cavities, especially in the mesenteric glands.

*Remarks.*—The best surgeons of the present day agree in the opinion, that when a tumour is ascertained to be cancerous—unless it be scirrhus, in its earliest stage—removal by the knife, so far from prolonging life, actually shortens it, especially if the dangers of the operation be taken into account. "Of 1172 patients, the subjects of cancer, not operated on, 18 lived more than thirty years after the first appearance of the disease; whilst of 801 operated on by excision or caustic, the existence of 4 only was prolonged for a similar time. 14 patients operated on, and 34 not operated on, lived from twenty to thirty years; and 88 in the first category, and 228 in the second, lived from six to twenty years after the first appearance of the disease."* While I am far from

* Cyclop. of Prac. Med., *Art. Scirrhus.*

desiring to dissent from the conclusion which must necessarily be drawn from these statistics, I believe that the operation performed in the case above detailed was justifiable. The position of the tumour; its rapid growth, producing great pain from distension of the surrounding integuments alone; the rapid absorption of the bone, giving rise speedily to perforation, pressure upon the brain, and death—together with the earnest desire of the patient to have it extirpated—seemed to justify a resort to the knife. And when it is remembered, that from the puncture of exploration there had sprouted out a vascular fungus, which bled upon the slightest touch, the operation was imperiously called for. It is true the patient was 73 years old; but then, her system had not been materially weakened, and she was in a better condition to undergo an operation than most persons are at fifty. I have no doubt but that her life was lengthened one or two months, at least, and that she suffered less pain than she would have suffered without the operation.

This case presents another instance of the value of anesthetic agents in surgical operations. The ether undoubtedly saved the patient from a shock, which, considering her age and temperament, might have been fatal. The length of time, too, that she was subjected to the influence of the agent, without the supervention of a single unpleasant symptom, argues strongly in favour of its harmlessness when properly exhibited. I do not believe the chloroform, the effects of which I have frequently witnessed, would have acted so pleasantly in this case as the ether—nor do I like it as well as the ether, under any circumstances.

Where did the tumour originate? within the cranium or external to it? We can merely speculate upon this subject. It is not impossible to conceive of a morbid growth within the cavity of the cranium developing itself to such an extent as gradually to perforate the bone and show itself externally, without producing fatal lesion of the cerebral mass. It is well known that the root of a tree will run beneath a stone wall, and as it increases in size, will lift the wall without making any perceptible impression upon the soft earth beneath. And in the case before us, we have seen the hard and apparently unyielding internal table of the scull give way before the expansion of an artery. Reasoning, therefore, from analogy, it would not be impossible to believe that the tumour commenced in the dura mater.

In opposition to this, however, it may be stated, that, on the first of December, when the tumour was large and fluctuating in every part, firm pressure upon it did not disturb the functions of the brain; that at the time the operation was performed, the perforation in the bone was not at all in proportion to the size of the tumour; that the pericranium formed the *base* of the cyst, instead of covering the upper portion of the tumour only, as would have been the case had the disease originated internally; that the patient had never suffered from headache until the tumour was as large as a walnut; and, that the occipital and temporal arteries—*external vessels*—contributed chiefly to the nourishment of the tumour. Add to this the fact, that when first observed it was very small, which is almost incompatible with its internal origin. Instead of a small, round, distinct tumour, there would first have appeared upon the scalp a slight elevation, undefined in its outlines, which by gradually increasing would at last have shown itself in full relief. Had it originated internally, it would have been perfectly immobile, but we learn that from the first it could be pushed about, to some extent, beneath the skin.

From a review of the whole case, I conclude, that the disease originated in the pericranium, and that if the operation had been performed before the bone was much involved, or the system became tainted, there would have been no return of the disease.

I am indebted to my friend Dr. Wm. J. Moore for the notes taken at the autopsy; and to my pupil and kinsman, Mr. T. W. Upshur, for valuable aid in the application of the dressings throughout the treatment of the case.

*Norfolk, (Va.,)* July 6th, 1848.

